# Morphology and Rheology of a Cool-Gel (Protein) Blended with a Thermo-Gel (Hydroxypropyl Methylcellulose)

**DOI:** 10.3390/foods11010128

**Published:** 2022-01-05

**Authors:** Zhili Ji, Long Yu, Qingfei Duan, Song Miao, Hongsheng Liu, Wangyang Shen, Weiping Jin

**Affiliations:** 1 Cereal Engineering, School of Food Science and Technology, Wuhan Polytechnic University, Wuhan 430023, China; whwangyangshen@126.com (W.S.); jwpacademic@outlook.com (W.J.); 2 Center for Polymer from Renewable Resources, School of Food Science and Engineering, South China University of Technology, Guangzhou 510640, China; felyu@scut.edu.cn (L.Y.); 202010105344@mail.scut.edu.cn (Q.D.); liuhongsheng@scut.edu.cn (H.L.); 3 Sino-Singapore International Joint Research Institute, Guangzhou Knowledge City, Guangzhou 510663, China; 4 Teagasc Food Research Centre, Moorepark, Fermoy, P61 C996 Co. Cork, Ireland; Song.Miao@teagasc.ie

**Keywords:** gelatin, HPMC, gel, phase transition, compatibility

## Abstract

This study investigates the morphological and rheological properties of blended gelatin (GA; a cooling-induced gel (cool-gel)) and hydroxypropyl methylcellulose (HPMC; a heating-induced gel (thermo-gel)) systems using a fluorescence microscope, small angle X-ray scattering (SAXS), and a rheometer. The results clearly indicate that the two biopolymers are immiscible and have low compatibility. Moreover, the rheological behavior and morphology of the GA/HPMC blends significantly depend on the blending ratio and concentration. Higher polysaccharide contents decrease the gelling temperature and improve the gel viscoelasticity character of GA/HPMC blended gels. The SAXS results reveal that the correlation length (ξ) of the blended gels decreases from 5.16 to 1.89 nm as the HPMC concentration increases from 1 to 6%, which suggests that much denser networks are formed in blended gels with higher HPMC concentrations. Overall, the data reported herein indicate that the gel properties of gelatin can be enhanced by blending with a heating-induced gel.

## 1. Introduction

Gelatin, derived from the partial hydrolysis of collagen, has a wide range of application in the food, pharmaceutical, cosmetic, and photographic fields [1,2,3,4]. The food products made from gelatin gel are commonly consumed by children and adults due to their special texture and mouthfeel [5]. Considering its thermal reversible property, gelatin gel can melt at a relatively low temperature (melt-in-mouth), which renders it the preferred gelling agent in yoghurt products, sugar confectioneries, and the coating films of nuts [6,7,8,9,10]. Unfortunately, under certain conditions, the gel strength, gelling temperature, and gel thermal stability of gelatin might be too low. For example, the hot weather in summer destroys gelatin gels due to their weak gel thermal stability. As a result, the consumers of these gels may experience an unpleasant impression and poor taste. Moreover, the food shelf life will be shortened. Therefore, the question of how to improve the gelatinous properties of gelatin has attracted great attention, and research efforts have been focused on using other food hydrocolloids, such as starch and carrageenan, to improve these properties, particularly the thermostability of gelatin under relatively high temperatures.

Interestingly, the physiochemical properties of hydrogels can be improved simply by blending a cooling-induced gel (cool-gel) with a heating-induced gel (thermo-gel). This processing method is relatively easy and does not require any chemical crosslinking agent to enhance product performance, which renders it important on both scientific and commercial levels. The most popular and widely available thermo-gel used to improve the properties of hydrogels is hydroxypropyl methylcellulose (HPMC) [11]. In particular, HPMC improves the tensile properties of cool-gel films such as collagen [12], surimi [13,14,15], and starch [16,17,18,19,20,21]. Meanwhile, gelatin and hydroxypropylated starch, both of which are cool-gels, are commonly used to enhance the processibility and moisture permeability of HPMC [16,22,23]. In a study conducted on surimi protein-HPMC blending, Chen et al. [15] showed that the structural geometry of the combined gel matrix depends on the processing time, and that longer times lead to variations in rigidity, thermal stability, and gel strength. Moreover, HPMC enhances the mechanical strength and thermal stability of fish gelatin [24]. Liu et al. [25] used chemical mapping techniques to study the phase composition of composites of polysaccharides and proteins. Our previous study also studied the effect of pH gelation behavior and morphology of gelatin/HPMC blends, and found that the molecular conformation of gelatin chains was altered by the pH in the surrounding environment, subsequently leading to the changes in gel strength and stability of composite gels [26]. The unique phase transition behavior of the cool-thermo gel system provides a good opportunity to further explore the relationship between microstructures and the performance of polymeric materials, particularly hydrogels. Unfortunately, previous research has mostly focused on the influence of the HPMC additive on the rheological properties of gelatin (or gelatin product), and there is no systemic report on how phase separation and gelation behaviors affect gel performance.

It is well known that the properties of a blending system depend on its morphology, which is mainly controlled by compatibility and phase separation behavior. In a system containing two opposite thermo-reversible gels, compatibility and phase separation are complex and strongly dynamic. For example, the phase behavior of a gelatin/HPMC composite gel depends on the concentration, blending ratio, and temperature. Considering that HPMC is a thermo-gel and gelatin is a cool-gel, the gelation and phase separation behaviors of the gelatin/HPMC composite gel are expected to reach equilibrium at different temperatures. In this work, we focus on how the compatibility and phase behaviors of the cool-gel (gelatin) and thermo-gel (HPMC) affect their solution and gel properties, in particular the effects of the blending ratio and solution concentration. The phase diagram of the GA/HPMC blends was established based on blending ratio and solution concentration. Rheometers, fluorescence microscopes, and SAXS were used to characterize the blending system, and the relationship between microstructures and gel performance was determined.

## 2. Materials and Methods

### 2.1. Materials

Food-grade gelatin (type A, from porcine skin, Bloom 250) (GA) was supplied by Rousselot Company, Giangzhou, China, and pharmaceutical-grade hydroxypropyl methyl cellulose (HPMC) (29% methoxyl content on dry basis, and 8.45% hydroxypropyl content on dry basis) was purchased from Hopetop Pharmaceutical Company, Huzhou, China. Analytical-grade NaN_3_, NaOH, fluorescein 5(6)-isothiocyanate, and DMSO chemicals were bought from Aladdin, Shanghai, China, and they were used as received.

### 2.2. Preparation of Stock Solutions

GA and HPMC stock solutions of varying concentrations (2, 4, 6, 8, 10, 12, and 14 wt%) were prepared with a pH5 according to the previous study [26]. GA (different amounts) was first dispersed in cold deionized water and left to swell for 2 h. Then, the solutions were heated to 60 °C with continuous stirring to achieve complete dissolution. Before heating, 0.02% sodium azide (NaN_3_) was added to the GA solutions to inhibit the growth of microorganisms. As for the HPMC solutions, they were prepared by dispersing HPMC powder in hot water and stirring for 30 min. Afterward, the solutions were cooled to ambient temperature with continuous stirring to achieve complete dissolution. All the solutions were stored in a refrigerator at 4 °C prior to use.

### 2.3. Determination of Phase Diagram

To determine the phase diagram of the GA/HPMC system, a series of GA/HPMC blends of varying concentrations (1, 2, 3, 4, 5, 6, and 7 wt%) were prepared by mixing 0, 2, 4, 6, 8, 10, 12, and 14 wt% gelatin stock solutions with 0, 2, 4, 6, 8, 10, 12, and 14 wt% HPMC stock solutions, respectively. The solutions were mixed at 55 °C with a continuous stirring for 1 h. Subsequently, 5 g of each blended solution were transferred to plastic centrifuge tubes that were sealed to prevent water evaporation. The tubes were held in a water bath at 25 °C for 24 h to determine whether phase transition (from gel to liquid state) or phase separation would occur. The different phase behaviors of the mixed systems were recorded.

### 2.4. Microstructure Observation

The micro-networks of 6/0, 6/1 6/2, 6/3, 6/4, 6/5, and 6/6 GA/HPMC blends (labelled G6H0, G6H1, G6H2, G6H3, G6H4, G6H5, and G6H6, respectively, were observed using an IX 53 fluorescence microscope (Olympus, Tokyo, Japan) equipped with a color and monochrome camera (DP 73, Olympus, Tokyo, Japan). GA was covalently labelled with fluorescein isothiocyanate (FITC) before mixing with HPMC solutions. First, 10 mg of FITC were dissolved in 10 mL DMSO solution. Then, 100 μL of the FITC solution were added to 100 mL of the GA stock solution (5 wt%, pH 10). The mixture was allowed to react for 1 h at 40 °C with continuous stirring. Then, the reaction was stopped by adding ethanolamine. Finally, the mixture was dialyzed against deionized water for 48 h to remove unreacted FITC, and NaN_3_ (0.02 wt%) was added to inhibit the growth of bacteria. The deionized water was changed every 2 h during dialysis. The FITC-labeled GA was lyophilized, and the yellow powder obtained was mixed with HPMC. The sample solutions were poured onto glass slides, covered with cover slips, and hermetically sealed with oil, then they were observed at the excitation wavelength of 460–495 nm. The emission of samples was recorded between 510 and 550 nm.

### 2.5. Viscosity Measurements

A rheometer (HR-2 Discovery Hybrid Rheometer, Co. TA, New Castle, DE, USA) with a concentric cylinder cell (28 mm inner diameter) was used to investigate the gel property of GA/HPMC blends (G6H0, G6H1, G6H2, G6H3, G6H4, G6H5, and G6H6) by analyzing the variation of viscosity as a function of temperature. The solution was first heated from 25 to 85 °C at the rate of 2 °C/min. Then, the temperature was decreased back to 25 °C at the same rate. The rotor speed was set to 25 rad/s during the entire heating and cooling process.

A parallel-plate geometry with a diameter of 40 mm was used to measure the flow pattern and study the effect of shear rate on the viscosity of the pure and blending solution at 25 °C. The shear rate was increased from 0 to 1000 s^−1^ in 1 min.

### 2.6. Rheological Properties

#### 2.6.1. Temperature Sweep

The dynamic rheological properties were explored using small-amplitude oscillatory shear test. The frequency was set at 1 Hz and the strain at 1% (this value lies within the linear range of viscosity). To evaluate the effect of temperature on the mixed systems, temperature ramps were implemented at the scan rate of 2 °C/min, with heating from 5 to 85 °C, followed by holding at 85 °C for 5 min, then cooling back to 5 °C with the same scan rate of 2 °C/min. The samples were placed between parallel plates, and a small amount of silicone oil was spread over the periphery of each sample to prevent moisture evaporation. Changes in the storage modulus (G′) and loss modulus (G″) were recorded as a function of temperature. The gelation and melting temperatures of GA and HPMC are defined as the temperature at which G′ is equal to G″ (the G′/G″ crossover point) [22,23].

#### 2.6.2. Frequency Sweep

To assess the dynamic mechanical behavior of GA/HPMC blends, 6% gelatin and 6% HPMC were prepared according to Section 2.2, and then a dynamic frequency sweep was performed at 25 °C and 1% strain (this value lies within the linear range of viscoelasticity, which was determined by the strain sweep presented in Figure S1). Within the identified linear viscoelastic region, the frequency oscillated between 0.1 and 100 rad/s. Changes in storage modulus (G′) and loss modulus (G″) were recorded for H0–H6 blends, and the frequency-dependence of G′ was determined based on the following power law equation [27]:

G′ = Sω^n^ (1)

where S is the gel strength, ω is the angular frequency, and n is the viscoelastic exponent.

### 2.7. Small-Angle X-ray Scattering (SAXS)

A SAXSess small-angle X-ray scattering system (Anton-Paar GmbH, Glaz, Austria) equipped with a PW3830 X-ray generator (PANalytical) was used to study the gel structure. Measurements were performed according to a previously described method [28,29]. Samples were measured at 50 mA and 40 kV using a Cu Kα radiation source (λ = 0.1542 nm). Each gel sample was stored at 4 °C for 24 h prior to analysis. The data recorded using an image plate were analyzed by the IP Reader software with a Perkin Elmer storage phosphor system. All data were normalized, and the background and smeared intensities were removed using the SAXS quant 3.0 software for further analysis.

The relationship between q and θ was determined according to the following equation [30]:

q = 4πsinθ/λ(2)

where θ means the scattering angle.

## 3. Results and Discussion

### 3.1. Phase Diagram of GA/HPMC Blends

**Figure 1:**
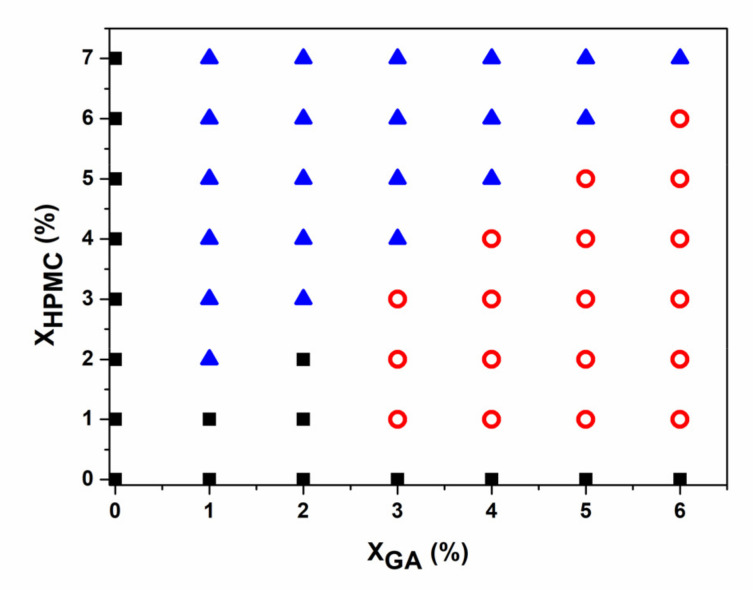
Phase diagram of the GA/HPMC blends quenched to 25 °C. ■ indicates a single liquid phase; **o** corresponds to a single gel system, and ▲ represents a phase separation system that includes a liquid phase (HPMC) as the top layer and a gel phase (GA) as the bottom layer.

Figure 1 shows the phase diagram of the GA/HPMC blends that were held at 25 °C for 24 h. It is shown that the compatibility and phase behavior of the mixed systems exhibited strong dependence on concentration and blend ratio. Moreover, the blends containing a very low polymer concentration yielded homogeneous solutions at 25 °C, and when the GA to HPMC ratio was less than 1, phase separation occurred. The gelatin-rich lower layer was in the gel state, whereas the HPMC-rich upper layer was in a liquid-like state. The blends composed of 3% gelatin formed yellow gels with no apparent (macroscopic) phase separation after storage at 25 °C. However, based on the transparency of the mixtures, microscopic phase separation occurred. Near the gelation temperature of gelatin, it is expected that the HPMC phase would be captured and immobilized by the gel network during phase separation, resulting in a uniform gel. To test this theory, mixtures containing 6 wt% GA and 1–6 wt% HPMC were tested.

### 3.2. Fluorescence Micrographs of Macrostructure

**Figure 2:**
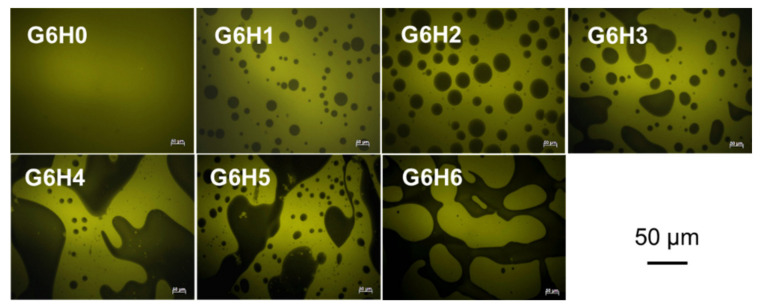
Fluorescence micrographs of GA/HPMC mixed systems observed at room temperature. The clear zones (yellow) correspond to the fluorescence of the labeled protein (covalently stained by FITC), and the dark ones correspond to the polysaccharide-rich regions.

The light and black zones that appear in the fluorescence micrographs shown in Figure 2 correspond to protein-rich and polysaccharide-rich areas, respectively. As expected, the micrograph of pure gelatin solution (G6H0) exhibited a uniform light color. However, the proportion of light to dark areas in the micrographs corresponding to G6H1–G6H6 blends varied depending on the blending ratio. The diversity in the microstructures and phase-separated networks of the blended systems (Figure 2) is attributed to the incompatibility between the two biopolymers. The size of polysaccharide-rich areas and the degree of connection between them increased with increasing polysaccharide concentration. For a fixed gelatin concentration, the gel microstructure changed from emulsion-like (with the HPMC-rich phase being the dispersed droplet phase) (e.g., Figure 2 G6H1, G6H2, and G6H3) to bi-continuous (e.g., Figure 2 G6H4, G6H5) to extensive phase-separated networks, where the GA-rich phase clearly lost continuity (Figure 2 G6H6) as the HPMC concentration was increased.

The phase separation of the gels was induced by the immiscibility of the two polymers, similar to many other protein-polysaccharide systems [31,32,33]. Significant phase separation occurs when the gel microstructure exhibits a bi-continuous organization of the protein- and polysaccharide-rich phases, leading to phase organization at the interphase (continuous region between the separated phases where distinct particles can be observed). Gelation kinetics play an important role in capturing this particular microstructure and in the final organization of the polymer-rich phase.

### 3.3. Viscosity of Solutions

**Figure 3:**
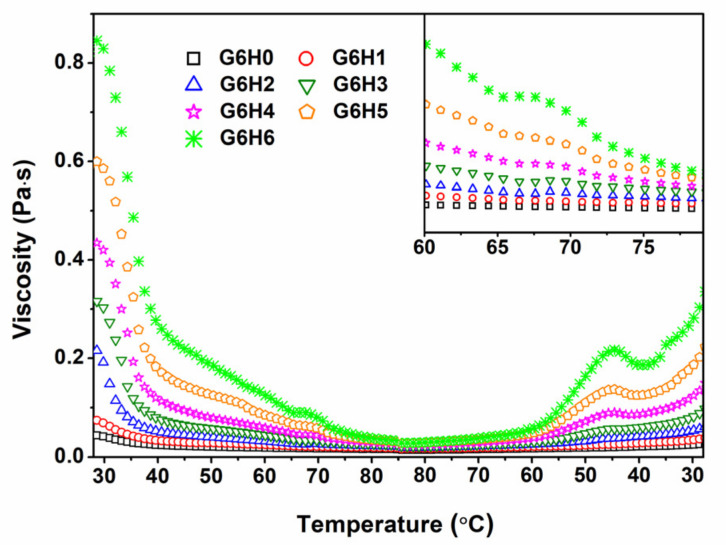
Effect of temperature on the viscosity of GA/HPMC mixed systems.

The viscosity of GA/HPMC solutions of varying blending ratios was studied at different temperatures and a specific shear rate. As shown in Figure 3, the viscosity of pure gelatin (G6H0) decreased with increasing temperature, as expected. Similar trends were observed for the GA/HPMC mixed systems, and the systems having higher HPMC contents exhibited larger viscosity values during both increasing and decreasing temperature regimes. At a fixed shear stress, the viscosity of GA/HPMC blends first decreased with increasing temperature due to the destruction of the gel network formed by gelatin molecules. Then, a slight shoulder (peak) was observed at ~65 °C for blends with HPMC contents ≥3% (see the enlarged part in Figure 3). This viscosity shoulder is attributed to the formation of a crosslinked network of HPMC molecules by the association of hydrophobic groups, on the one hand, and the association of hydroxyl groups, on the other hand, at high temperatures [22,34,35]. The peak viscosity value shifted to lower temperatures as the concentration of HPMC was increased. This was probably due to the increased interactions between HPMC molecules. The viscosity peaks observed during the cooling process were much more pronounced than those detected during heating, and they shifted to lower temperatures (~45 °C) due to the hysteresis [36] and/or condensation of chains at a lower temperature [23,34]. The final viscosity of the GA/HPMC blends after cooling was much lower than the initial viscosity measured at the beginning of the heating stage. The variation between the initial and final viscosity values is related to the fact that the blend solutions with no shear stress exhibit a 60 s equilibrium stage at 25 °C. Due to the absence of shear stress, the blend solution can form gels that are much stronger than those formed under shear stress, resulting in asymmetrical temperature-viscosity curves.

**Figure 4:**
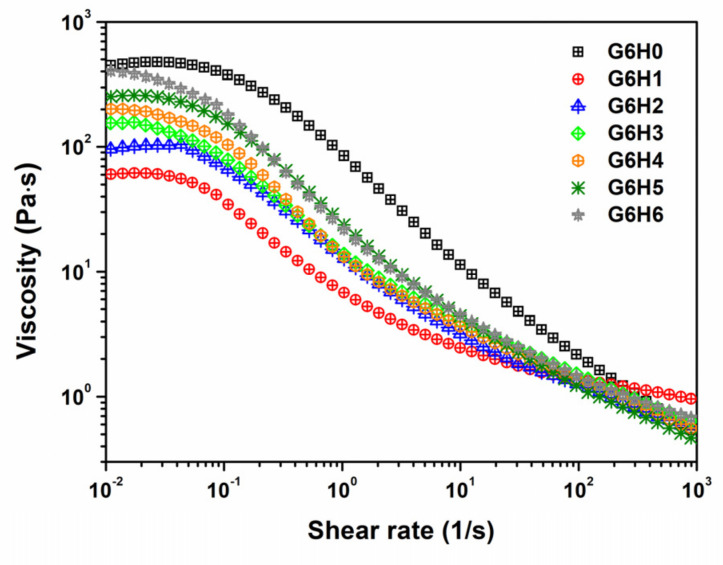
Variation of viscosity as a function of shear rate for the GA/HPMC mixed systems at 25 °C.

The effect of shear velocity on the viscosity of different blends at room temperature was also investigated. As shown in Figure 4, all GA/HPMC mixtures exhibited Newtonian behavior at low shear stress and pseudoplastic behavior at high shear rates. Under low shear stress, all the samples showed a Newtonian plateau before they reached a critical shear rate and their viscosity dropped, suggesting a dimensional stability related with a firm network formed by gelatin and HPMC chains. Moreover, the apparent viscosity of pure gelatin was higher than that of GA/HPMC mixtures, and the G6H6 system characterized by the highest polymer concentration exhibited the largest viscosity values among the investigated systems. Over the critical shear rate, the shear-thinning behavior observed under high shear rates was related to the disentanglement and molecule rearrangement of polymers under shear force, as described by Heyman et al. [37]. For pure gelatin, the shear-thinning behavior was stronger than that detected for GA/HPMC mixtures. This indicates that HPMC undermines the shear-thinning behavior of gelatin and narrows the stable-value region of the blended samples in the low-shear stage.

### 3.4. Rheological Properties of GA/HPMC Blends

#### 3.4.1. Gelling and Melting Temperatures

**Figure 5:**
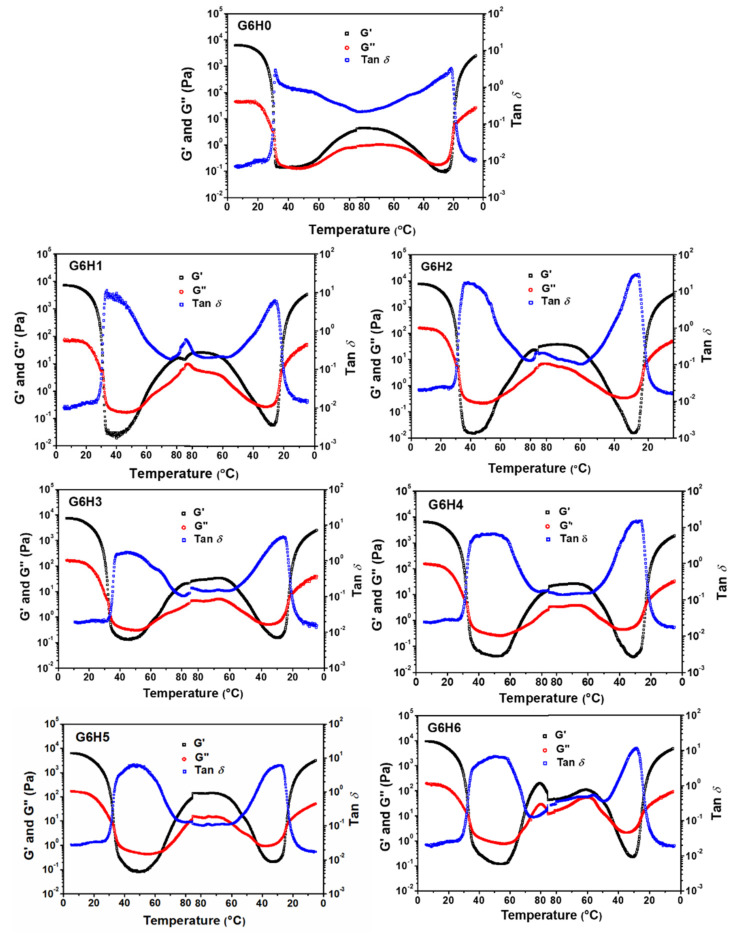
Variation of storage modulus (G′), loss modulus (G″), and tan δ as a function of temperature for solutions of GA and GA/HPMC blends.

Figure 5 presents the storage modulus (G′), loss modulus (G″), and tan δ profiles recorded during the gel formation of GA and GA/HPMC blends (heating and cooling profiles). The gel transition point of gelatin was detected at a relatively low temperature. However, HPMC led to thermal gels. All samples formed gels that were heat-reversible [22,38]. During the first plateau, the G′ values of GA and GA/HPMC systems were two orders of magnitude higher than the corresponding G″ values. This indicates that strong gels were formed due to the building of triple helix networks between gelatin, and HPMC molecules were trapped in them. The values of G′ for GA decreased with increasing temperature up to 40 °C, which suggests that the gelatin network transitions from gel to sol. The decrease in G′ was accompanied by an increase in tan δ, meaning that the elastic character of the gel also decreased. Due to the association of hydrophobic groups, on the one hand, and hydroxyl groups, on the other hand, all HPMC-containing blends gradually formed a crosslinked network as the temperature was increased to 85 °C. The formation of the gel network was confirmed by the occurrence of G′ and G″ plateaus with higher values of G′. By comparing our results (Figure 5) to those of previously published gel formation studies of pure systems, it may be concluded that the thermally incompatible GA and HPMC components in the blends form their own networks, resulting in a phase-separated mixed gel [39]. The FTIR spectra presented in the study of Liu et al. [25] further verify this conclusion.

The sol-gel transition points (G′ = G″) for the GA/HPMC blends are listed in Table 1. Each system was characterized by two gelling points and two melting points that correspond to the melting temperature of the gelatin gel (T_mg_) and the gelling temperature of HPMC (T_gh_) in the heating stage, and the melting temperature of the HPMC gel (T_mh_) and the gelling temperature of gelatin (T_gg_) in the cooling stage. The sol-gel transition points of gelatin and HPMC were ~30 and ~56 °C, respectively. Based on the data listed in Table 1, the addition of 1–6% HPMC increased the melting point of gelatin (31–33 °C) compared to the pure GA sample (T_mg_ = 31 °C), showing a slight increase. This may be due to the fact that the protein aggregation induced by HPMC improved the gel network, which can only be destroyed by high energy. Moreover, the increase in T_mg_ value at higher HPMC content is likely associated with the higher local protein concentration resulting from biopolymer phase separation [40], as described in Figure 2. Higher HPMC contents also lead to greater entanglement between HPMC and GA chains, which strengthens the gel network. Finally, the increase in total solution concentration is another factor that may be responsible for improving gel network. As for the gelling temperature of HPMC (T_gh_), it was lower for the G6H0, G6H1, and G6H2 samples than for the G6H4, G6H5, and G6H6 samples. The increased Tgh at higher HPMC contents may be attributed to the complete phase separation between GA and HPMC and the relatively low viscosity of HPMC at these conditions. These phenomena promote the self-aggregation of HPMC molecules, resulting in increased local HPMC concentration and lower gelling temperatures. In the cooling stage, hysteresis [41] and/or condensation of chains occurs at lower temperatures [22,23]. Therefore, G′ and G″ crossover (tan δ = 1) is shifted to lower temperatures, resulting in reversed sequences of gel-sol regions. The increase in melting temperature implies that HPMC addition improves the thermal stability of gelatin gels.

The increase in the gelation temperature and thermal stability of gelatin in GA/HPMC systems compared to the GA system is related to phase distribution (Figure 2) and local polymer concentration. Each one of the two regions produced upon phase separation is rich in one biopolymer but depleted in the other. This causes an increase in the local concentration of the predominant biopolymer. One may assume that the self-association of abundant GA chains in definite areas of the protein-rich phase contributes to the rheology of the mixed systems.

#### 3.4.2. Dynamic Mechanical Properties

**Figure 6:**
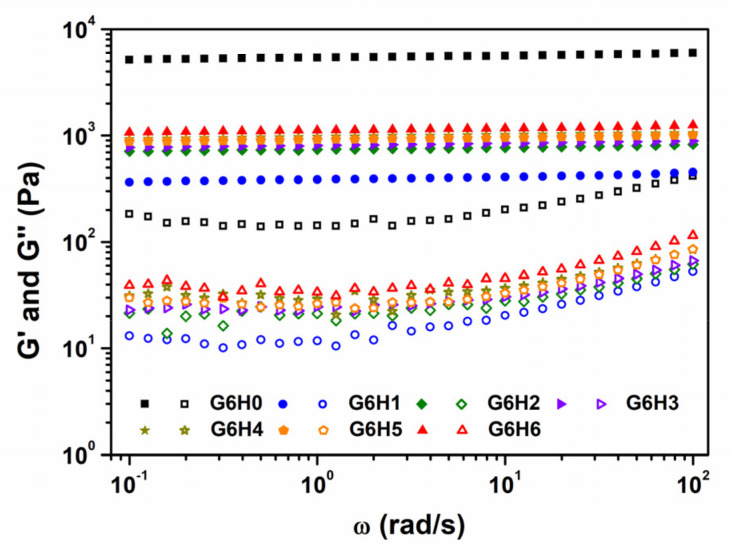
Storage modulus (G′) and loss modulus (G″) variation as a function of frequency for GA and GA/HPMC mixed gels at 25 °C. The closed and open symbols correspond to G′ and G″, respectively.

Frequency sweep analyses were conducted on the co-hydrogels at room temperature (25 °C), in the frequency (ω) range of 0.1–100 rad/s. The G (ω) versus ω plots shown in Figure 6 bear the characteristic signature of the gel concerned. The storage modulus G′ is a measure of the elastic energy stored in the network. Therefore, higher G′ values are indicative of stronger elastic networks. Herein, all investigated samples exhibited typical solid-like behavior (G′ > G″), irrespective of the frequency, and G′ values did not vary markedly with frequency. The gelatin gel formed in the presence of HPMC was obviously weaker than that formed in the absence of the polysaccharide. This suggests that HPMC disturbs the continuity of the gelatin network (shown in Figure 2) and thus decreases its strength. During the period of gel formation, the loss modulus G” increased, indicating that the viscous part of the system increased. It means that the viscoelasticity of gelatin gels increases in the presence of HPMC, whereas the rigidity decreases. Meanwhile, the melting temperature of GA/HPMC gels were higher than that of pure gelatin gel (seen in Table 1). The interaction between GA and HPMC increased the thermal stability of gelatin gel on one hand. On the other hand, the stiffness of gelatin was decreased.

The variation in storage modulus at low frequencies follows the power law given by G′ = Sω^n^. The n and S parameters are both sensitive to the crosslinking density inside the gel, with higher S values signifying “harder” gels. The data presented in Table 1 show that n was close to 0 for all investigated samples, which confirms their solid-like behavior [42]. The S value of pure gelatin was much larger than the values of GA/HPMC blends, which suggests that pure gelatin behaves more like a solid than the blends. Moreover, the S of GA/HPMC blends increased from 385 to 1119, with increasing HPMC content from 1 to 6%. This increase may be attributed to the effect of increased biopolymer concentration in promoting the entanglement of GA and HPMC chains. It may also be attributed to the sharp microscopic phase separation observed at higher HPMC contents, which leads to larger local biopolymer concentrations. Therefore, after quenching to below the gelation temperature of GA, a stronger, more stable gel network was formed, with a higher melting temperature for gelatin at higher HPMC contents.

### 3.5. SAXS Analysis of Phase Separation

**Figure 7:**
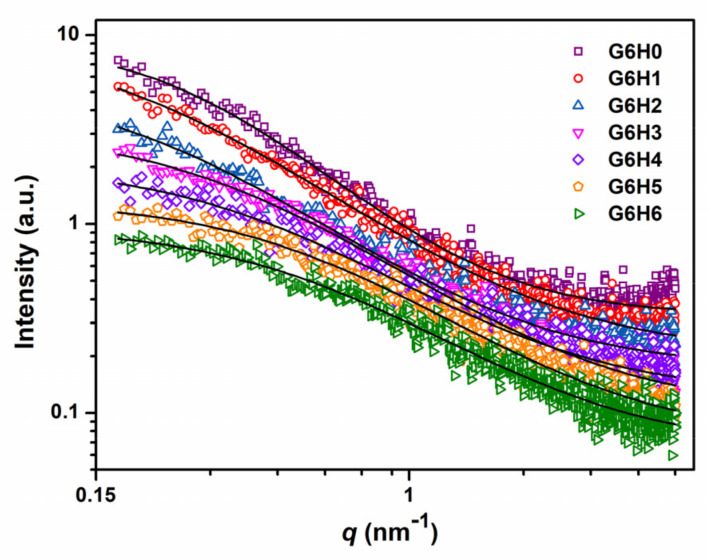
SAXS profiles of different GA/HPMC blended gels. The data are fitted based on the correlation length model.

To investigate the structural differences between the GA/HPMC hydrogels investigated herein, their SAXS profiles were recorded at room temperature. The gel network of each sample was determined based on the viscosity measurements and rheology analysis. As shown in Figure 7, the recorded profiles exhibited clear differences, and the highest value of scattering intensity as observed for the pure gelatin gel (G6H0). Comparatively, the SAXS scattering intensities of GA/HPMC composite gels were very small, and they decreased with increasing HPMC content. This indicates that the HPMC addition reduced the electron density difference between the gel clusters and the matrix. Similarly, Guestaw et al. [43] showed that the SAXS scattering intensities of WPI-xanthan gum blended gels decreased upon the addition of xanthan gum to WPI, which suggests that the protein globules of the gel undergo aggregation. The scattering profiles of GA/HPMC hydrogels were fitted across the entire q-range using the correlation length model equation [44]:

(3) $$I\left(q\right)=\frac{I\left(0\right)}{1+{\left(\xi q\right)}^{m}}+\mathrm{bkg}$$

where I(0) is the scattering intensity extrapolated to the scattering vector q = 0, and ξ is the correlation length that describes the distance at which adjacent polymer chains tend to interact (ξ reflects the density of the tangled network in a semi-dilute polymer solution). The Porod exponent, m, provides information regarding the local polymer structure [44,45]. Based on the values summarized in Table 2, the correlation length of pure gelatin was 3.66 nm, and the length of the GA/HPMC mixed gels decreased from 5.16 to 1.89 nm with increasing HPMC concentration from 1 to 6%. The correlation length of G6H1 (5.16) and G6H2 (4.80) were larger than that of G6H0 (3.66), which may have been due to the interruption of gel network connectivity under the low HPMC contents. However, a much denser network was formed at higher HPMC contents (G6H3, G6H4, G6H5, and G6H6). On the one hand, the formation of a denser network can be attributed to protein molecule aggregation. On the other hand, it may be attributed to the interaction between GA and HPMC, such as the chain entanglement, which lead to the decreased correlation length. The Porod exponent, m, of the GA/HPMC mixed gels ranged between 1.60 and 1.64, which agrees well with the typical values determined for physical gels [46].

## 4. Conclusions

In this work, the gelation and phase behaviors of blended gelatin (cool-gel) and HPMC (thermo-gel) systems were assessed by analyzing the phase diagram of these blends. The obtained results show that the behavior of binary GA/HPMC systems changes from water-in-water emulsion-like to a bi-continuous network whose microstructure clearly depends on the ratio of the two polymers. The formation of phase-separated networks is attributed to the incompatibility between gelatin and HPMC, as well as to their reversed gelation behavior. The solid character of the mixed gels is correlated with the component constituting the continuous phase, and the gel stability of gelatin gels has no significant changes with increasing HPMC concentration. Based on the SAXS results obtained herein, larger HPMC concentrations lead to structural changes in GA/HPMC mixed gels at various length scales. These structural differences probably induce variations in rheological properties. The elucidation of GA/HPMC interactions is a key element in formulating and controlling the quality of manufactured foods, as well as in developing novel food processes and edible film materials.

## Figures and Tables

**Table 1:** Melting temperatures, gelling temperatures, n, and S of GA and GA/HPMC blends.

System	Heating Stage		Cooling Stage		Frequency Sweep	
	T_mg_ (°C)	T_gh_ (°C)	T_mh_	T_gg_	n	S
G6H0	30.71 ± 0.16 ^a^	-	-	20.06 ± 0.54	0.020 ± 0.000	5400 ± 5
G6H1	31.29 ± 0.37	56.18 ± 0.18	37.85 ± 0.13	22.49 ± 0.34	0.027 ± 0.001	385 ± 1
G6H2	32.08 ± 0.19	58.79 ± 0.12	41.54 ± 0.49	22.73 ± 0.27	0.021 ± 0.001	740 ± 1
G6H3	32.68 ± 0.29	58.60 ± 0.20	40.70 ± 0.23	22.92 ± 0.36	0.019 ± 0.000	810 ± 0
G6H4	33.10 ± 0.40	64.45 ± 0.08	43.96 ± 0.31	23.33 ± 0.12	0.019 ± 0.000	970 ± 0
G6H5	33.20 ± 0.19	65.88 ± 0.23	43.37 ± 0.44	24.13 ± 0.32	0.019 ± 0.000	915 ± 1
G6H6	33.51 ± 0.11	65.89 ± 0.22	42.54 ± 0.14	25.32 ± 0.17	0.021 ± 0.000	1119 ± 1

^a^ Mean ± standard deviation; (-) texture unsuitable for measurements.

**Table 2:** Fitted SAXS parameters of GA and GA/HPMC blending gels.

System	*ξ* (nm)	*m*
G6H0	3.66 ± 0.10 ^d^	2.01 ± 0.04 ^b^
G6H1	5.16 ± 0.28 ^f^	1.60 ± 0.03 ^a^
G6H2	4.80 ± 0.34 ^e^	1.62 ± 0.05 ^a^
G6H3	3.06 ± 0.10 ^c^	1.61 ± 0.03 ^a^
G6H4	2.63 ± 0.14 ^b^	1.60 ± 0.05 ^a^
G6H5	1.90 ± 0.06 ^a^	1.64 ± 0.05 ^a^
G6H6	1.89 ± 0.06 ^a^	1.63 ± 0.05 ^a^

Different lowercase letters within each column indicate significant differences (*p* < 0.05) among the different groups.

## Data Availability

The data presented in this study are available on request from the corresponding author.

## Supplementary Materials

The following supporting information can be downloaded at: https://www.mdpi.com/article/10.3390/foods11010128/s1, Figure S1: Strain sweep curves of (a) 6% GA and (b) 6% HPMC solutions recorded at 1 Hz and 25 °C. The solid and hollow symbols represent G′ and G″, respectively.
