# Supplementary material for: Morphology and Rheology of a Cool-Gel (Protein) Blended with a Thermo-Gel (Hydroxypropyl Methylcellulose)
Source: Foods. 2022 Jan 5;11(1):128. doi: 10.3390/foods11010128 (PMC8750888; doi:10.3390/foods11010128)
Supplement: Supplementary file 1 [file foods-11-00128-s001.zip › foods-1512920-supplementary.pdf]

Supplemantary Materials

This supporting information file contains the following:

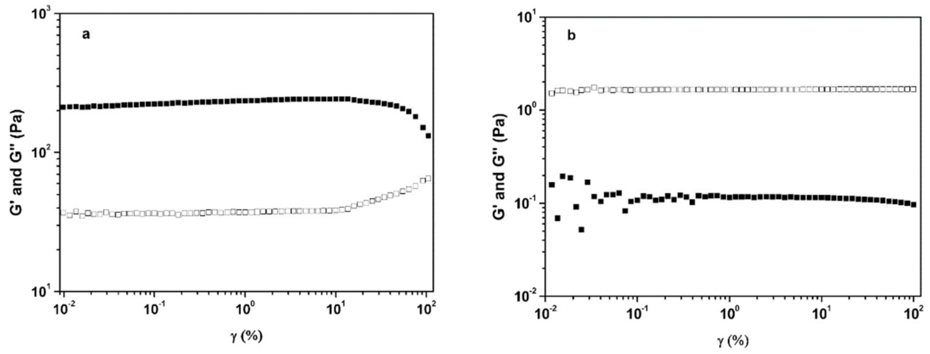

Figure S1. Strain sweep curves for 6% gelatin (a) and 6% HPMC (b) solutions at a frequency of 1 Hz at 25 °C. The solid and hollow symbols represent G′ and G″, respectively.
